# A novel machine learning approach for surface roughness quantification and optimization of cast-on-strap lead-antimony alloy via two-point correlation function

**DOI:** 10.1038/s41598-023-39619-z

**Published:** 2023-08-17

**Authors:** Mohamed Basyoni, Yang Jiao, Nageh K. Allam

**Affiliations:** 1 Materials Science and Engineering Department, Arizona State University, Tempe, AZ USA; 2 Energy Materials Laboratory, Physics Department, School of Sciences and Engineering, The American University in Cairo, New Cairo, 11835 Egypt; 3 German Co. for Manufacturing Batteries, New Salheya, Egypt

**Keywords:** Energy science and technology, Engineering, Materials science, Physics

## Abstract

Surface roughness has a negative impact on the materials’ lifetime. It accelerates pitting corrosion, increases effective heat transfer, and increases the rate of effective charge loss. However, controlled surface roughness is desirable in many applications. The automotive lead-acid battery is very sensitive to such effects. In our case study, the cast-on-strap machine has the largest effect on the surface roughness of the lead-antimony alloy. In this regard, statistical correlation functions are commonly used as statistical morphological descriptors for heterogeneous correlation functions. Two-point correlation functions are fruitful tools to quantify the microstructure of two-phase material structures. Herein, we demonstrate the use of the two-point correlation function to quantify surface roughness and optimize lead-antimony poles and straps used in the lead-acid battery as a solution to reduce their electrochemical corrosion when used in highly corrosive media. However, we infer that this method can be used in surface roughness mapping in a wide range of applications, such as pipes submerged in seawater as well as laser cutting. The possibility of using information obtained from the two-point correlation function and applying the simulated annealing procedure to optimize the surface micro-irregularities is investigated. The results showed successful surface representation and optimization that agree with the initially proposed hypothesis.

## Introduction

**Figure 1:**
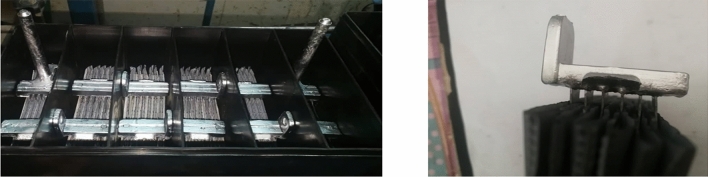
Lead-acid battery poles and strap. (Credits: German Co. for manufacturing batteries).

The crown jewel of materials science is the materials tetrahedron. Knowing those important relations between its components is the key to design new materials with the desired properties. Surface characteristics can help to predict the failure of materials. In the lead acid batteries industry, poles and straps (connectors between each electrochemical cell) are important for electric and thermal current connection in the battery^1^. Therefore, controlling the onset of the surface cracks in such materials is crucial to enhance the battery performance and manufacturing processes as well as help in the design of lower mass lead battery components, thus reducing lead consumption and toxicity. Poles and straps are shown in Fig. 1. While poles are the terminals of the battery, the straps connect the positive plates with each other and the negative plates with each other in each single 2.1 V battery cell to form a 12.6 V battery stack^2^. During the welding process of the plates to each strap, the plates’ lugs are fluxed and welded producing a highly rough surface. Battery straps are immersed in a corrosive medium (sulfuric acid of 1.27–1.28 sp. gr)^2^. Rough surfaces influence the electrochemical corrosion of the materials, resulting in poor electrical conductivity, thermal conductivity, and initiation of fatigue cracks during operation^3–5^. We have empirically observed that many battery poles exploded under high rates of discharge. Roughness is the main influence parameter for general corrosion that is investigated widely in other materials systems in the literature. However, in our system, producing poles and connectors between cells with the roughened surface can do the same as other metal systems increasing corrosion, cavity paths may be produced due to gaseous embrittlement leading to propagation of surface cracks towards the core. While the battery is operating, continuous hydrogen and oxygen gasses elevate by the chemical reaction, vibrations of the battery also can lead to fatigue if there were initial cracks influenced by corrosion. In high progressive corrosion samples, larger diameter cavities and corrosion paths are found on the poles may also lead to pole explosion influenced by surface shrinkages limiting any attempt to enhance the cyclability of the battery on high progressive corrosion.

Poles and straps are made of lead-antimony (Pb-Sb) alloy, which has a wide freezing range. At the instant that the component is being solidified in the cast-on-strap machine, the liquid phase of the Pb-Sb alloy has lower density than the solid phase, leading to shrinkage of the grains during solidification^2^. Open shrinkage appears on the surface as pitting, which impacts the corrosion rate and current conduction. Upper open shrinkage that is connected to the car battery, terminals loose connection and cause terminal fusion at high discharge currents. Lower open shrinkage on parts immersed in diluted H_2_SO_4_ or at the path of hydrogen and oxygen evolution can initiate stress corrosion cracking, leading to fatigue fracture of poles or straps^6^. Closed shrinkage appears as cavities that reduce the effective thermal and electrical conductivity or even fusion of battery poles and strap fracture in extreme conditions. Shrinkage can be controlled by optimizing the operation conditions, such as pouring temperature, mold temperature, water cooling temperature, cooling water flow rate, and design of risers and runners of the mold^7^. Positive plates’ corrosion, water loss, and negative plate sulfation issues were investigated intensively in the literature, with many solutions recommended^2^. Despite all those investigations, the corrosion of straps has not been extensively investigated despite the fact that it could be a common reason of failure, especially upon the use of 2D additives to enhance the battery cycle life as in modern automotive lead-acid battery^8^. This type of corrosion crack propagates under the stress of vibrations during battery operation.

In order to analyze and predict surface roughness effect on different materials, there are many approaches that were found in the literature, which were generally applied to cutting machines as a logical main controller of surface roughness. The first class of approaches are the Machining Theory Approaches (MTA). These approaches depend on the working principle of the surface-producing machines such as cutting tools The working principle and machines operating conditions are taken into account to produce a geometrical model using a computer-aided design (CAD) tool to predict the surface roughness. For instance, chip-cutting machines. The mathematical model is based on the molecular mechanical friction theory and Hencky-Ilyushin’s plasticity theory. Unfortunately, this approach can be easily affected by any mechanical change. Depending on the cutting tool conditions, parameters, geometry, and relative motion between the work piece and cutting tool. Assumptions of surface topography simulation were put in to get successful quantification and prediction of surface profile. Despite the accuracy of the machining theory approach models, they are not comprehensive and require a lot of complex factors that may contribute to the surface roughness to be considered and the machine should be in its optimum conditions which is not the case for longer life working machines^9^.

Another class of approaches is the Experimental Investigation Approach, it is often used when there is no clear relationship between the causes and effects of the surface profile. It relies upon the researcher’s understanding of what actually happens on the material. In this approach, vibration or acceleration signals are fed to an analyzer that produces ASCII files. Cutting speed, depth of cut, feed rate, and approach angle of cutting are important parameters that needed to be taken into account in the surface analysis. This approach is manageable, depending on the depth of understanding of materials engineering phenomena, and the results are accurate. However, it is not comprehensive and specific to particular machines, and too many factors need to be considered^9^.

The Designed Experiments Approach has a statistical systematic experimental approach in which repetition of the experiment is required to acquire sufficient data to be analyzed. At first, experimental parameters (factors affecting the surface roughness) such as cutting depth, cutting velocity, etc. are determined. Two-level factorial experiment is designed and applied to collect data. We try to preserve the path of the steepest ascent by choosing a reference factor and using it as a standard to draw the appropriate path for each factor of the experiment. Then we run trials and make sure that there is no deviation from the steepest ascent path. If the response yields no-substantial improvement, a three-level factorial design is needed to keep the path of the steepest ascent with a good response. Stationary points conditions shall be recorded. The designed experiments approach helped in determining which machine parameters affect surface roughness the most and the influence of the cutting tool and the workpiece materials. However, it only shows the parameter to response relation and takes a lot of time to collect data but it was the onset to develop first-order and second-order models^9^.

At last, the Artificial Intelligence Approach or as widely known by the AI approach is the most promising approach in surface analysis and optimization. By simulating how the human mind can process information and make decisions, many systems and algorithms have been developed. The most known is the Artificial Neural Network (ANN). ANN is based on many assumptions, they are defined as simple elements that process information, transmit signals over connection links, each connection link has associated weights that multiply the transmitted signals, and the output signal is determined by employing an activation function to the incoming signal of each neuron^9^. Feed-forward ANN is a typical ANN in which the connection between nodes does not form a complete cycle. The inputs are multiplied by weights and then added together to get a sum of weighted input values. If the sum is below the threshold, the output value is − 1, and if above it, the value is 1. This simple architecture is helpful when many individual ANNs are necessary to collect data and then added together to result in a cohesive output. By using simple programming, we can deal even with incomplete data to get accurate results. In the work of Deshpande et al.^10^ they have introduced an ANN modeling for Inconel 718 alloy using untreated and cryogenically treated carbide inserts. Cutting parameters, sound, force, and vibration factors were used to foretell surface roughness with an accuracy of up to 98%. Despite the high accuracy of their model, in many machining cases where surface roughness is vitally important, a lot of factors shall be introduced to be a general technique for every machinery^10^.

In our model, the quantification and optimization method of the surface is a general model, we have used it for the Pb-Sb material system in the lead acid battery industry. It offers an evaluation of the component after machining, requires no data during operation (finished sample), is compliant with different material systems as it does not depend on mechanical parameters and the surface optimization is very accurate regardless of the machining conditions.

To this end, statistical correlation functions are microstructure descriptors that can be used to implement intelligent technology in many industrial applications. In general, the most common representation is the standard $n$-point correlation function $S_{n}$ as n expands from 1 to ∞. It is known as the probability of finding *n* points or events of materials that can be used to quantify heterogeneous materials, polycrystalline materials, and directional bonding materials. Correlation functions were significantly used to predict the effective properties of such materials^11,12^. Heterogeneous materials are usually composed of different phases. Herein, we introduce the use of a statistical two-point correlation function, widely used to quantify binary alloys, to study the surface roughness of Pb-Sb alloy. The idea depends on the fact that those alloys have two phases and the surface texture has troughs at a distinct height as revealed by the atomic force microscopy imaging. In other words, surface troughs can be translated into domains of two-phase material.

## Material and methods

**Figure 2:**
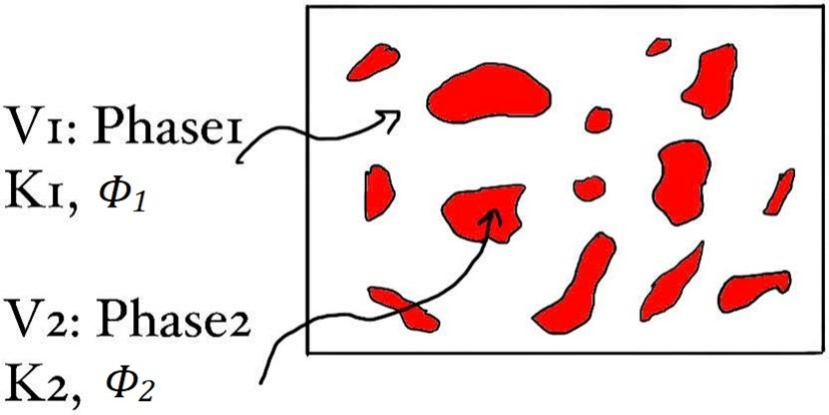
Schematic of a random two-phase material.

Consider a two-phase heterogeneous material (e.g., a binary alloy) consisting of phase 1, a region Ѵ_1_ of volume fraction ϕ_1_ with a general property coefficient K_1_, and phase 2, a region Ѵ_2_ of volume fraction ϕ_2_ = (1−ϕ_1_) with a general property coefficient K_2_. Both phases are static and independent of time as we assume that Ѵ_1_ ∪ Ѵ_2_ = Ѵ and Ѵ_1_ ∩ Ѵ_2_ = 0. Because the properties depend on the structure, K_1_ and K_2_ can be coefficients of any property (mechanical, chemical, electrical, …, etc.), see Fig. 2.

Let the probability of any point *x* (in phase 1) is $I^{\left( 1 \right)} \left( x \right)$:

1 $$I^{{\left( 1 \right)}} \left( x \right) = \left\{ {\begin{array}{*{20}l} {1,} & {x \in \nu } \\ {0,} & {x \in otherwise} \\ \end{array} } \right.$$

${I}^{(1)}\left(x\right)$ is called phase indicator. In other words, if we computationally throw an arbitrary point ${I}^{(1)}\left(x\right)$, it will be equal to 1 if it is located at phase 1 and 0 otherwise. Same for phase 2, $I^{\left( 2 \right)} \left( x \right)$ is the phase indicator of phase 2 and

2 $$I^{{\left( 2 \right)}} \left( x \right) = \left\{ {\begin{array}{*{20}l} {1,~} & {x \in \nu 2} \\ {0,~} & {x \in otherwise} \\ \end{array} } \right.$$

Since phase 1 and phase 2 are independent of each other and complementary, i.e., ϕ_2_ = (1−ϕ_1_):

3 $${ } I^{\left( 1 \right)} \left( x \right) + I^{\left( 2 \right)} \left( x \right) = 1{ }$$

In general, the phase indicator function is

4 $$I^{{\left( i \right)}} \left( x \right) = \left\{ {\begin{array}{*{20}l} {1,} & {x \in \nu i} \\ {0,} & {x \in otherwise} \\ \end{array} } \right.$$

As we mentioned, phase $\user2{i }$ can be solid, fluid, or void. The surface/interface indicator function is

5 $${\mathcal{M}}\left( x \right) = \left| {\nabla I^{\left( 1 \right)} \left( x \right)} \right| = \left| {\nabla I^{\left( 2 \right)} \left( x \right)} \right|$$

For the probability functions in materials design for a given material domain distribution or periodic base cell can be represented as number of finite elements considering periodic boundary conditions. Statistical functions and reconstruction can find the effective and optimal distribution of those domains or phases (solids, liquids, voids) such that the objective function is minimized. As for the first step, we shall capture information about the microstructure using the correlation functions. Then, we apply Monte Carlo to reconstruct domain and get the information of the reconstruction via correlation function^11^. Digitized image pixels can be used to identify various properties, such as distribution of electric or magnetic fields, variation in physical properties of the medium, structure geometry, velocity fields, and temperature velocity. As a new dimension, here we introduce their use to identify surface irregularities.

### Two-point correlation function $\left( {{\varvec{S}}_{2} } \right)$

The two-point correlation function can be defined as:

6 $${\varvec{S}}_{2}^{{\left( {\varvec{i}} \right)}} = {\varvec{I}}^{{\left( {\varvec{i}} \right)}} \left( {{\varvec{x}}_{1} } \right){\varvec{I}}^{{\left( {\varvec{i}} \right)}} \left( {{\varvec{x}}_{2} } \right)$$

It is one of the widely used statistical microstructure descriptors. It is the probability that two random points $x_{1}$ and $x_{2}$ are positioned in the same phase. For statically homogeneous and isotropic media, two-point correlation functions depend only on the distance $r = \left| {x_{1} - x_{2} } \right|$. When the two points coincide (i.e., $r = 0$), the two-point correlation function is treated as a one-point correlation function and is equal the volume fraction of phase $i \to S_{2}^{\left( i \right)} \left( 0 \right) = \phi_{i}$. In a two-phase material, the relation between the two-point correlation of phases is defines as:

7 $${\varvec{S}}_{2}^{\left( 2 \right)} = {\varvec{S}}_{2}^{\left( 1 \right)} \left( {\varvec{r}} \right) - 2\phi_{1} + 1$$

The associated autocovariance function is defined as:

8 $$\chi \left( r \right) \equiv S_{2}^{\left( 1 \right)} \left( r \right) - \phi_{1}^{2} = S_{2}^{1} \left( r \right) - \phi_{1}^{2}$$

One crucial condition of $S_{2}^{\left( i \right)}$ for a two-phase homogenous material with dimensions *d* is that the d-dimensional Fourier transform of $\chi \left( r \right)$ shall be non-negative for all of the wave vectors $k,$ i.e., the spectral function is positive semidefinite.

9 $$\tilde{\chi }\left( k \right) = \smallint \chi \left( {\varvec{r}} \right)e^{{ - i{\varvec{k}}.{\varvec{r}}}} dr \ge 0, \;for\; all\,\, {\varvec{k}}$$

$\tilde{\chi }\left( k \right)$ is proportional to scattered radiation intensity.

For all ${\varvec{r}}$, the two-point correlation functions must satisfy the condition $0 \le {\varvec{S}}_{2}^{{\left( {\varvec{i}} \right)}} \left( {\varvec{r}} \right) \le \phi_{{\varvec{i}}}$ hence, the corresponding autocovariance function is given by:

10 $$- {\text{min}}\left( {\phi_{1}^{2} , \phi_{2}^{2} } \right) \le \chi \left( {\varvec{r}} \right) \le \phi_{1} \phi_{2}$$

As for homogeneous and isotropic media (i.e., $S_{2}^{\left( i \right)} \left( {\varvec{r}} \right)$ depend on the relative distances), the derivative $r = 0$ must be negative for all $0 < \phi_{i} < 1$:

11 $$\left. {\frac{{dS_{2}^{\left( i \right)} }}{dr}} \right|_{r = 0} = \left. {\frac{d\chi }{{dr}}} \right|_{r = 0} < 0$$

One more condition for statistically homogeneous media is,

12 $$S_{2}^{\left( i \right)} \left( r \right) \ge S_{2}^{\left( i \right)} \left( s \right) + S_{2}^{\left( i \right)} \left( t \right) - \phi_{i}$$

where $$r = t - s$$.

As we understand the nature of the two-point correlation function, we can obviously find out that the limits of $S_{2}$ can be expressed as:

13 $$\mathop {\lim }\limits_{r \to 0} S_{2} \left( r \right) = \phi_{1} \;and\; \mathop {\lim }\limits_{r \to \infty } S_{2} \left( r \right) = \phi_{1}^{2}$$

Generally, two-point correlation function is one of the most important and widely used probability functions to theoretically quantify morphological features of any material system. For homogeneous media, it can be acquired by randomly throwing line segments of length $r$ with a specific orientation and counting the fraction of times that $x_{1}$ and $x_{2}$ lie on the same phase^11^.

### Lineal-path function ${\varvec{L}}^{{\left( {\varvec{i}} \right)}} \left( {\varvec{r}} \right)$

**Figure 3:**
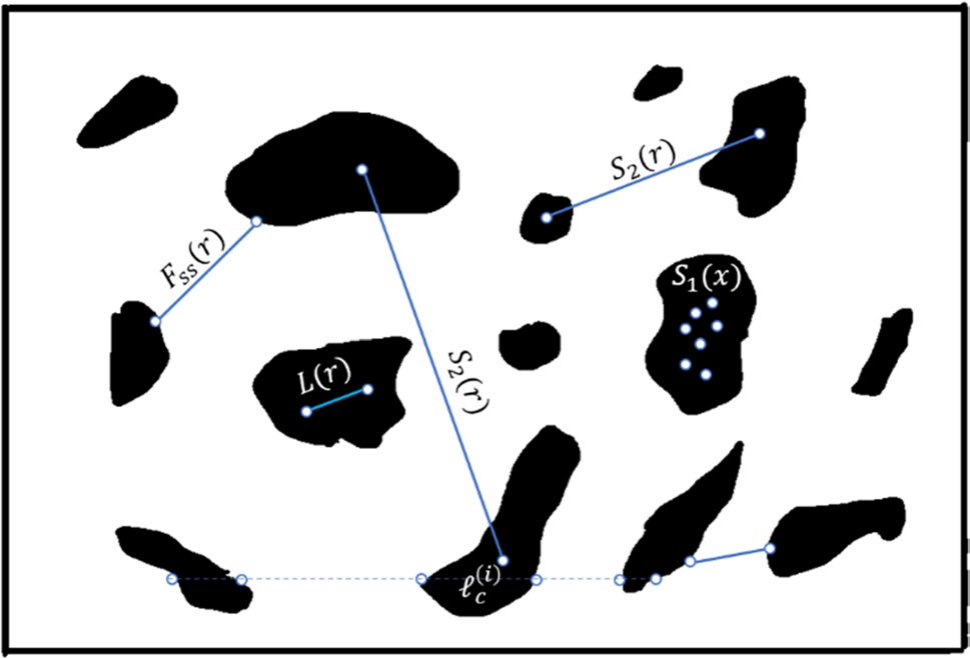
Different correlation functions representation in a two-phase material.

Lineal-path function is an appealing lower-order correlation function. For statistically homogeneous and isotropic media, it measures the probability that random line segment of length $\user2{r }$ lies entirely on the same phase of interest ${\varvec{i}}$ along ${\varvec{r}}$ direction. ${\varvec{L}}^{{\left( {\varvec{i}} \right)}} \left( {\varvec{r}} \right)$ contains information about linear partial, topological connectedness of the material microstructure, see Fig. 3. As ${\varvec{r}} = 0$, the lineal-path function shrinks on itself and can be treated as a probability of finding only one-point on the phase of interest (i.e., ${\varvec{L}}^{{\left( {\varvec{i}} \right)}} \left( 0 \right) = \user2{ }\phi_{{\varvec{i}}}$) and for ${\varvec{r}} \to \infty$ we have ${\varvec{L}}^{{\left( {\varvec{i}} \right)}} \left( \infty \right) = 0$. For homogeneous and anisotropic media, ${\varvec{L}}^{{\left( {\varvec{i}} \right)}} \left( {\varvec{r}} \right)$ will only depend on the magnitude of vector ${\varvec{r}} = {\varvec{x}}_{2} - {\varvec{x}}_{1} ,$ while it depends on the absolute positions ${\varvec{x}}_{1}$ and ${\varvec{x}}_{2}$ for inhomogeneous materials^11^.

For our sample, we have directly got our sample from cast-on-strap machine, before that the alloy of Pb–Sb could be produced by refining Pb to 99.985% and then alloyed with Sb to get the alloy composition shown in Table 1. Large scale samples are not ideal samples, where processing can extremely alter the materials properties. Many problems, in most of materials produced at the industrial scale, have not been widely investigated in the literature. This is just because at the lab scale, we usually use the ideal conditions just to eliminate the undesired problem to keep focus on the main ideal properties. On the other hand, researchers may just stop at this point and do not give attention to the actual problems that may appear during manufacturing.

**Table 1:** Chemical composition (wt%) of cast-on-strap alloy.

Element	Sb	Sn	As	Cu	Pb
Wt %	3.1	0.19	0.24	0.03	Bal

**Figure 4:**
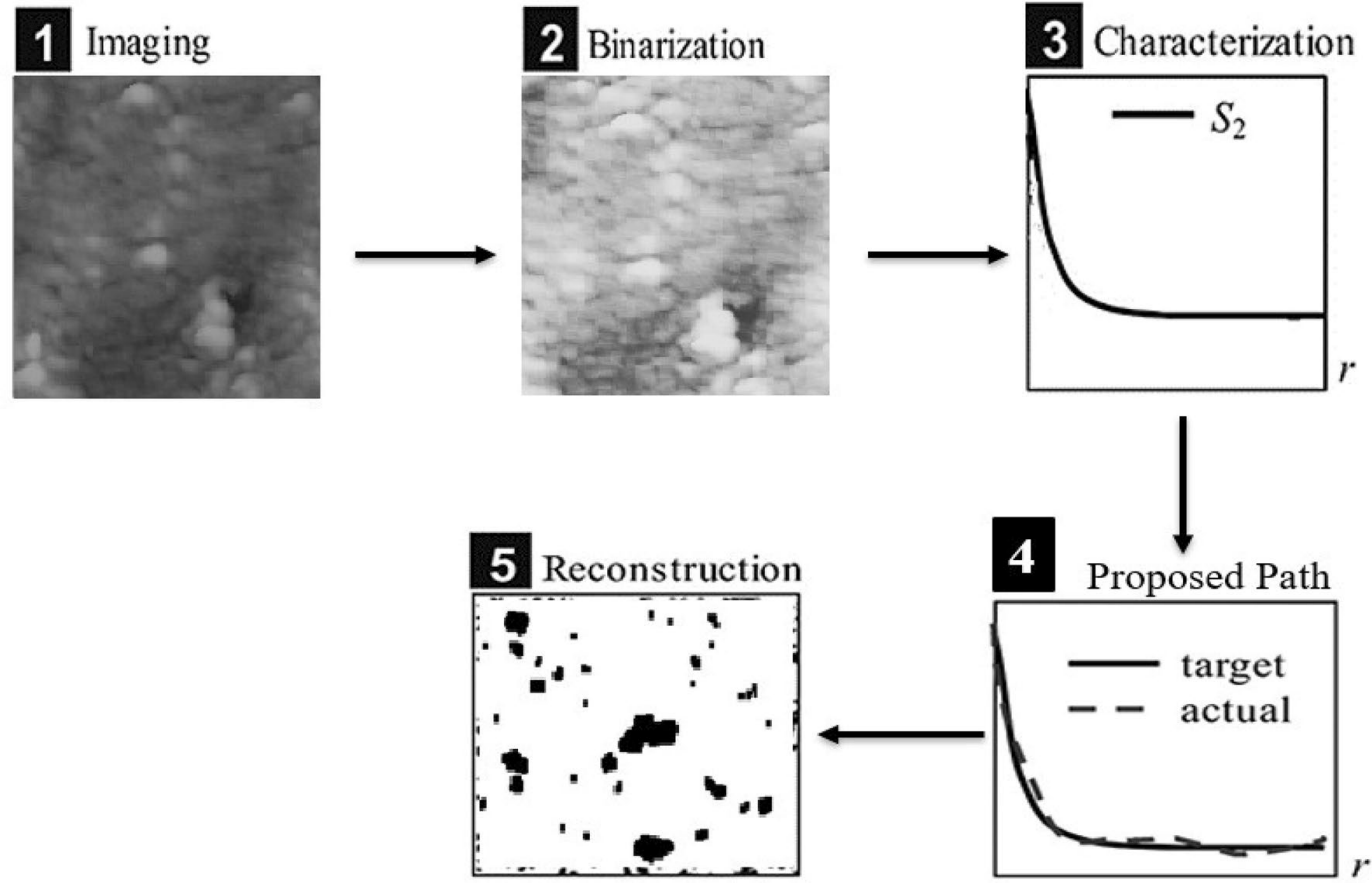
Schematic illustration of general steps of materials quantifications.

To illustrate the processing conditions at the cast-on-strap (COS) machine, the main parts have to be functionally automated, see Fig. 4. A furnace at temperature 470 °C is used to melt the alloy. Then, the melted alloy is pumped through pipes to the main mould with specific speed and being poured to the mould cavities within 2.2 s or depending on the design of each battery type. The mould has a water-cooling system with constant flow rate and constant water temperature not reaching 120 °C but in most cases, the cooling temperature is 110 °C for 8 s. The sample was taken from the furnace, quenched in water for 8 s and is cut into pieces, then its surface was polished to be prepared for imaging.

## Results and discussion

### Quantifying the surface roughness of the Pb–Sb alloy

**Figure 5:**
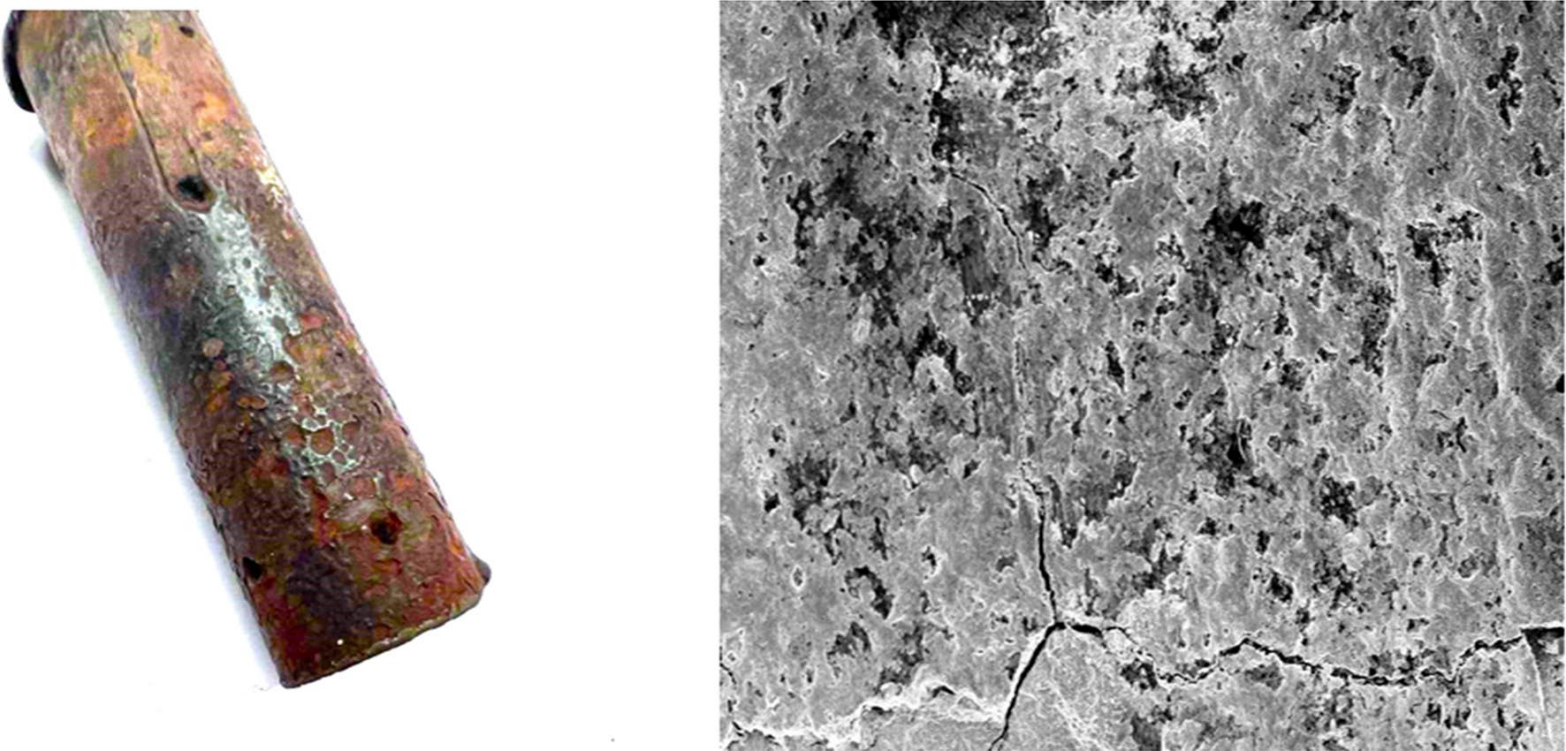
Corroded Lead-acid battery pole operated for 8 months, approximately 10,870 miles, and an SEM image showing crack initiations at 20 µm scale. (Credits: German co. for manufacturing batteries).

**Figure 6:**
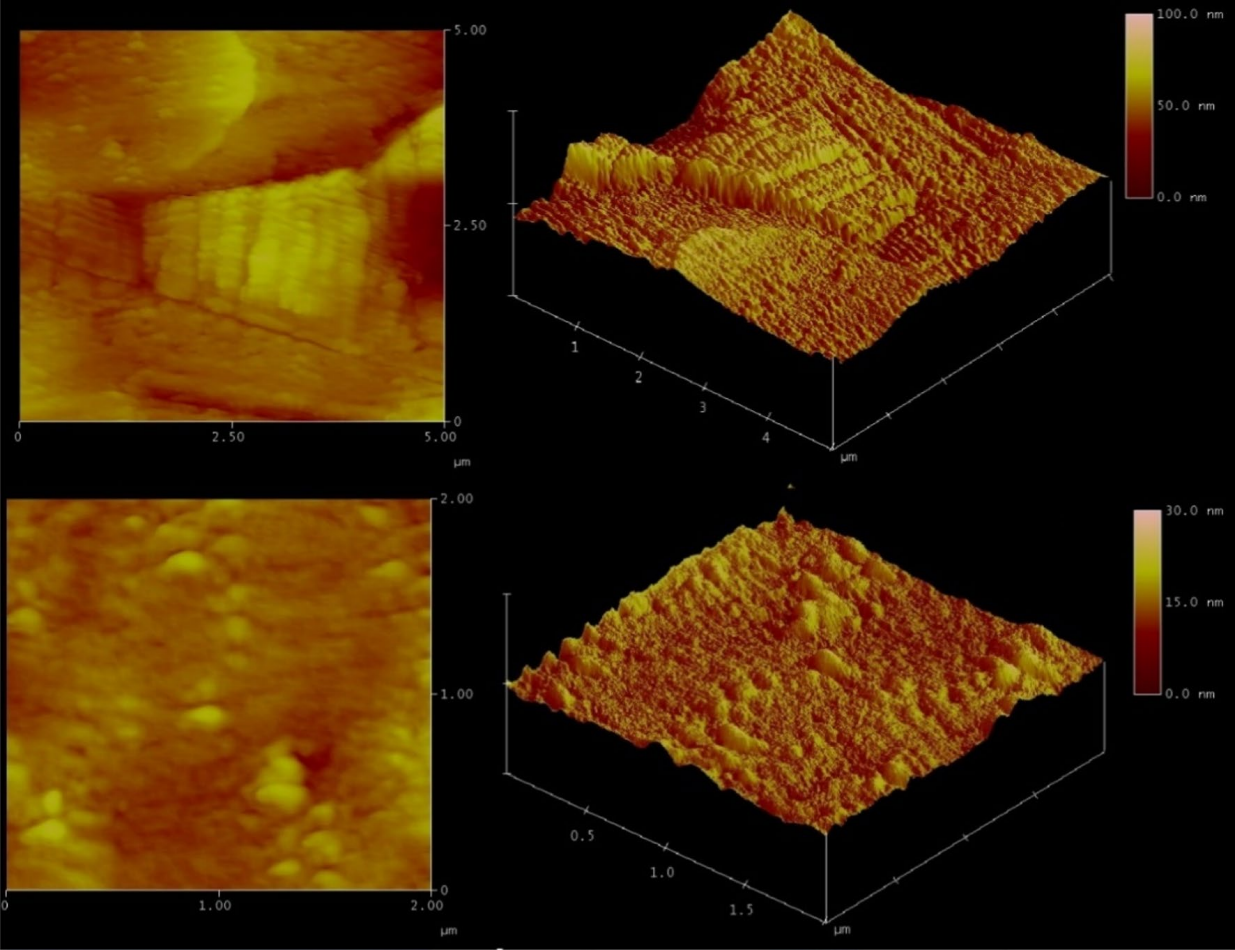
AFM images of the surface topography of Pb–Sb sample at 100 nm.

**Figure 7:**
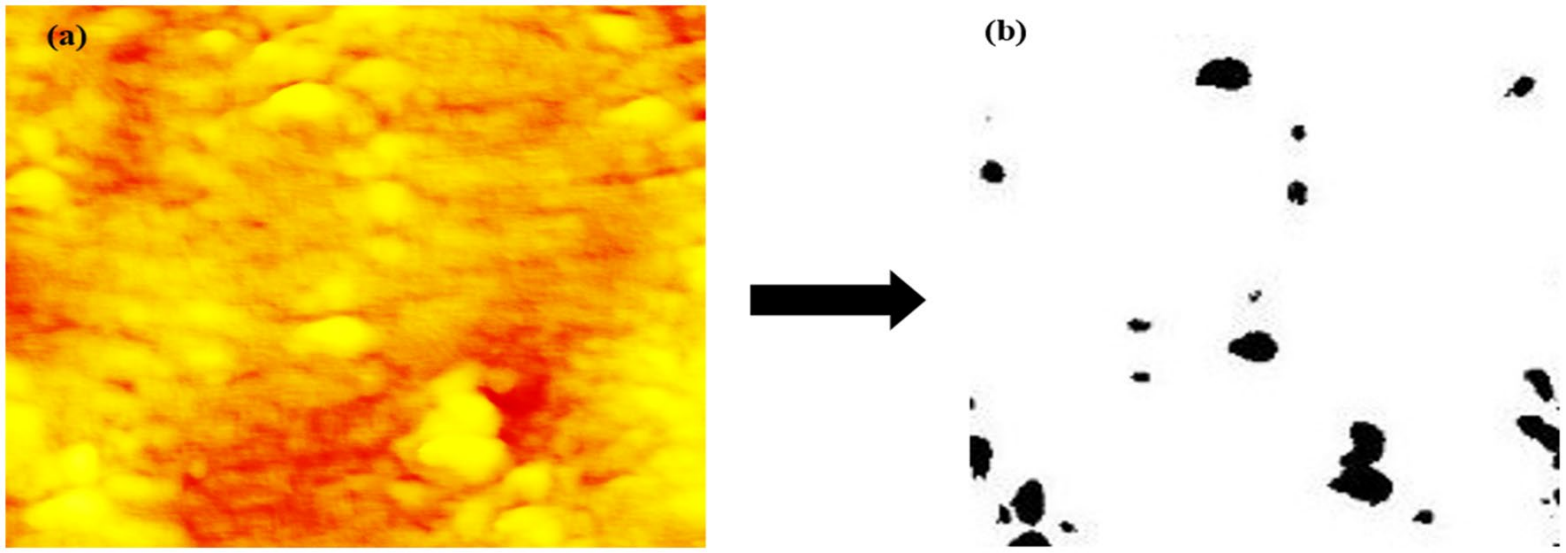
Binary image of surface topography of Pb-Sb sample with $\phi_{{\text{i}}} = 0.038$.

Our interest is to determine the surface characteristics of the Pb–Sb alloy, including roughness and surface defects that act as active sites to advance the corrosion of the Pb–Sb alloy during operation. Figure 5 shows a corroded lead-acid battery pole operated for 8 months, along with an SEM image showing crack initiation. Moreover, atomic force microscopy (AFM) images are shown in Fig. 6. The AFM image was converted into a binary greyscale image using MATLAB. The images were processed using an in-house C^++^ code to extract the data from the binary images and produce probability maps for the structure. In the programming code, the black pixels were set as the feature data of interest while the white pixels represent the matrix data, see Fig. 7. A binary image with distinct threshold was used to eliminate the multitude of spatial scales that can be convoluted on the grayscale images of the surface. This provides a clear representation of peaks and valleys on the surface texture and makes it available to use lower-resolution images for computation purposes.

The characteristics of the structure were computed using a combination of two-point correlation function $S_{2} \left( r \right)$ and lineal-path function $L\left( r \right)$. A surface image of a slice of Pb-Sb cast-on-strap sample is shown in Fig. 7b, where the black areas indicate higher surface toughness spots, which is the phase of interest, and the white areas show the equal roughness heights solid phase.

For simplification, only square binary images of length MAXX were used in the characterization, with MAXX being an even number. Note that the two-point correlation function for statistically homogeneous medium can be interpreted as the total length of line segment as well as its both ends lie completely on the same phase. In sampling $S_{2}$, we computed the probability of the fraction of times that distances between a black pixel $i$ and all other surrounding pixels $j$ of black pixels successfully separated by distances $r$ such that the end point is located at pixels centers to the total number of throwing line segments trials. Sampling the two-point correlation function only along the principal directions of hypercubic lattice, mainly in rows or columns in 2-D. Hence, $S_{2}$ is a linear function of distances between the adjacent pixels^13^.

14 $$S_{2} \left[ r \right] = \left( {1 - f} \right)S_{2} \left( i \right) + fS_{2} \left( {i + 1} \right) ;\;\;i \le r < i + 1,$$

where $f$ is the fractional part of $i$.

Lineal-path function $L\left( r \right)$ presents the probability of finding an entire line segment of length $r$ at the phase of interest. Sampling $L\left( r \right)$ is straight forward, we detect a point A at an oriented line at orthogonal direction and move A along this line until we encounter other phases at point B. Then, we calculated the ratio between lines of lengths with equal distance between A-B and total number of all inserted lines with all lengths. Consider line segment lengths at orthogonal directions are stored in an array counter $L\left[ r \right],$ while $r \le r_{i}$ an integer value increasing by 1 and $r_{i}$ is the length of $i{\text{th}}$ line.

15 $$L\left( r \right) = \frac{L\left[ r \right]}{{Lines \;with\; all\; possible\; lenghts }}$$

**Figure 8:**
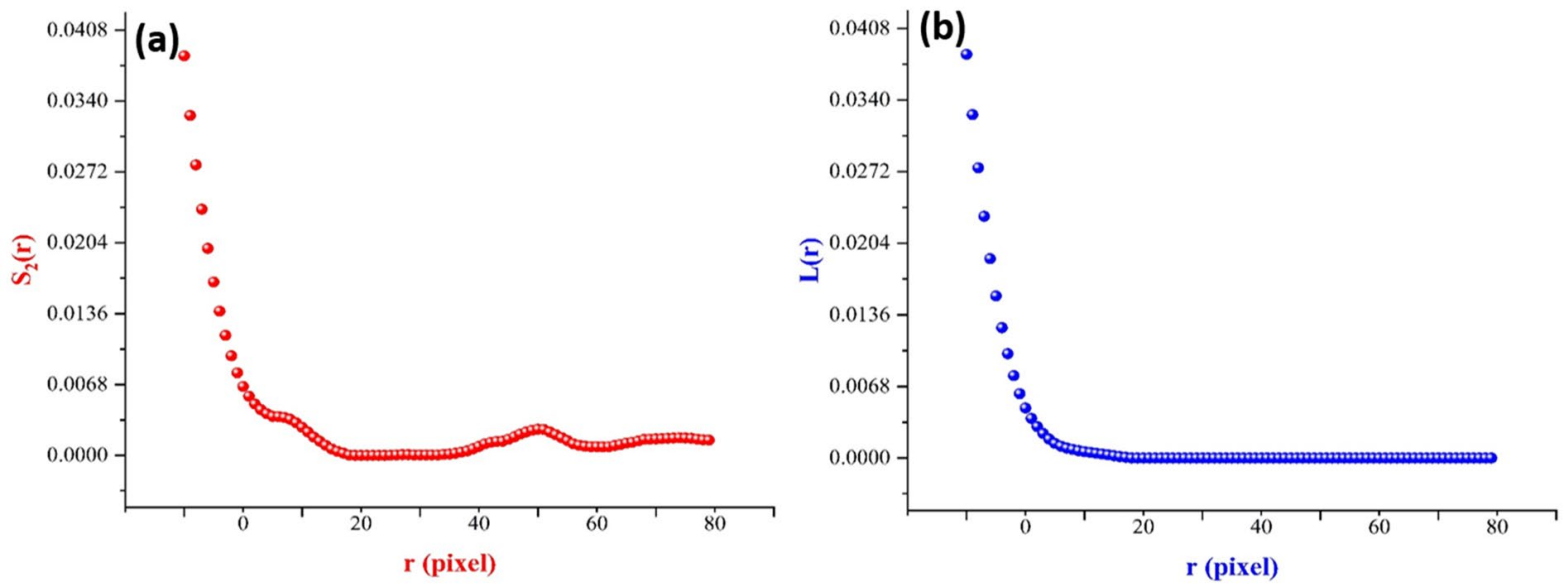
Data collected upon the use of the (**a**) two-point correlation function and (**b**) lineal-path function for surface topography of Pb–Sb sample with approximate $\phi_{{\text{i}}} = 0.038$.

Upon applying the code to the digitized image shown in Fig. 8, the surface structure probability functions were obtained. The information contained in $S_{2} \left( r \right)$ and $L\left( r \right)$ was similar, showing the volume fraction of the highly roughened part of the sample slice $\phi_{1} = 0.03836$ and the smooth surface with a volume fraction $\phi_{2} = 0.96164,$ see Fig. 8.

Regarding the data collected from $S_{2}$, the first portion of data exhibits almost no fluctuations, revealing that almost all line segments are at distances as $r \le 23$ pixels. This means that the larger part of the surface volume fraction with the same roughness height have diameters $r \le 23$. Also, as the $S_{2} \left( 0 \right)$ connection 2 points within the same phase and $L\left( 0 \right)$ is the line within the same phase as the spatial distribution $S_{2} \left( 0 \right)$ at small $r$ values may equal to the line pathways $L\left( 0 \right)$ as they are not highly convoluted at small *r* but highly convoluted for large $r$. Considering the binary image at Fig. 8b, $\phi_{i} = 0.038$ is very reasonable, meaning that our approach in characterizing the surface roughness of such alloy via correlation functions is successful.

### Surface reconstruction using simulated annealing

Simulated annealing method is used to assess the surface reconstruction. It is usually employed to solve such large-scale optimization problems. Also, it can be used to switch digitized image pixels to identify the optimum microstructure^14,15^. The superiority of the simulated annealing technique can be ascribed to the fact that it does not need special complex setups, inexpensive, and capable of scabbing local minima by accepting locally unfavorable configurations. One can predict the lowest possible energy state via simulated annealing based on the fact that; when a system is heated to high temperature $T$(excitation energy state), by slowly cooling down the system to temperatures near or equal to absolute zero, it samples all different energy states until equilibrium at the ground energy state (minimum stable energy state)^12,13^. For canonical ensemble, at a temperature $T$, the probability for the system to be at energy state $E$ is a Boltzmann distribution:

16 $$P\left( E \right) = Const* e^{{ - \frac{E}{T}}}$$

**Figure 9:**
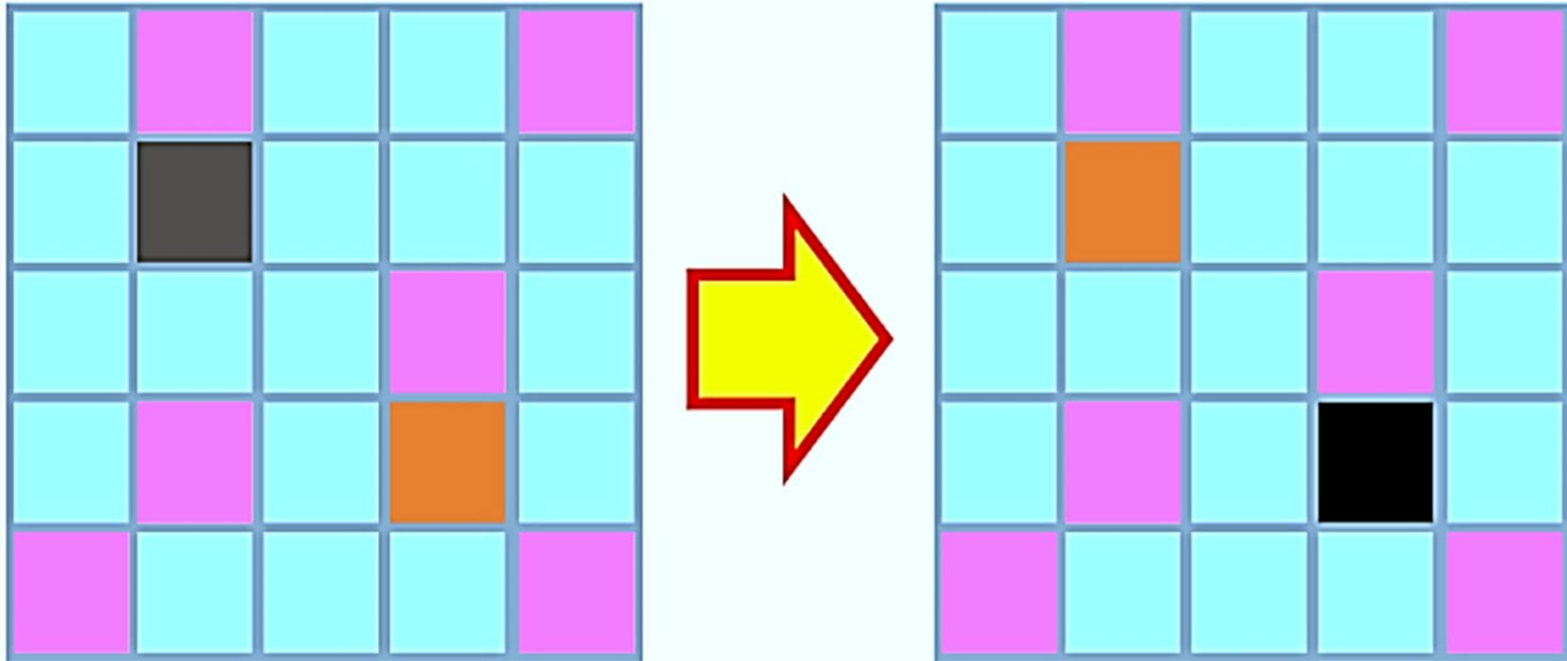
Visual representation of pixel swapping or exchange procedure to generate new microstructure from old one.

For each annealing step $t = k$, the system samples and reaches equilibrium temperature $T_{k}$. The temperature is then lowered for each annealing step $T\left( t \right)$ until it approximates the ground energy level. Thus, in the simplest form starting with given microstructures, states of two arbitrary pixels of different phases are swapped, conserving the volume fraction of both phases as shown in Fig. 9.

Change in energy between two successive states is calculated as:

17 $$\Delta E = E^{\prime} - E$$

Whether the new energy state is accepted as the next energy state or not is determined by the acceptance probability, which is given by

18 $$p\left( {\Delta E} \right) = \left\{ {\begin{array}{*{20}l} {1~,~} & {\Delta E \le 0,} \\ {e^{{ - ~\frac{{\Delta E}}{T}}} ,} & {\Delta E > 0,} \\ \end{array} } \right.$$

where T is a hypothetical high initial temperature.

Although, ideal annealing to reach the ground state is to decrease temperature steps according to $T\left( k \right)\sim \frac{1}{\ln \left( k \right)}$, it may cause very slow energy convergence. Hence, we use faster annealing schedule of

19 $$\frac{T\left( k \right)}{{T\left( 0 \right)}} = \lambda^{k} ;\;\;\lambda \to 1$$

where $\lambda$ is the annealing rate.

**Figure 10:**
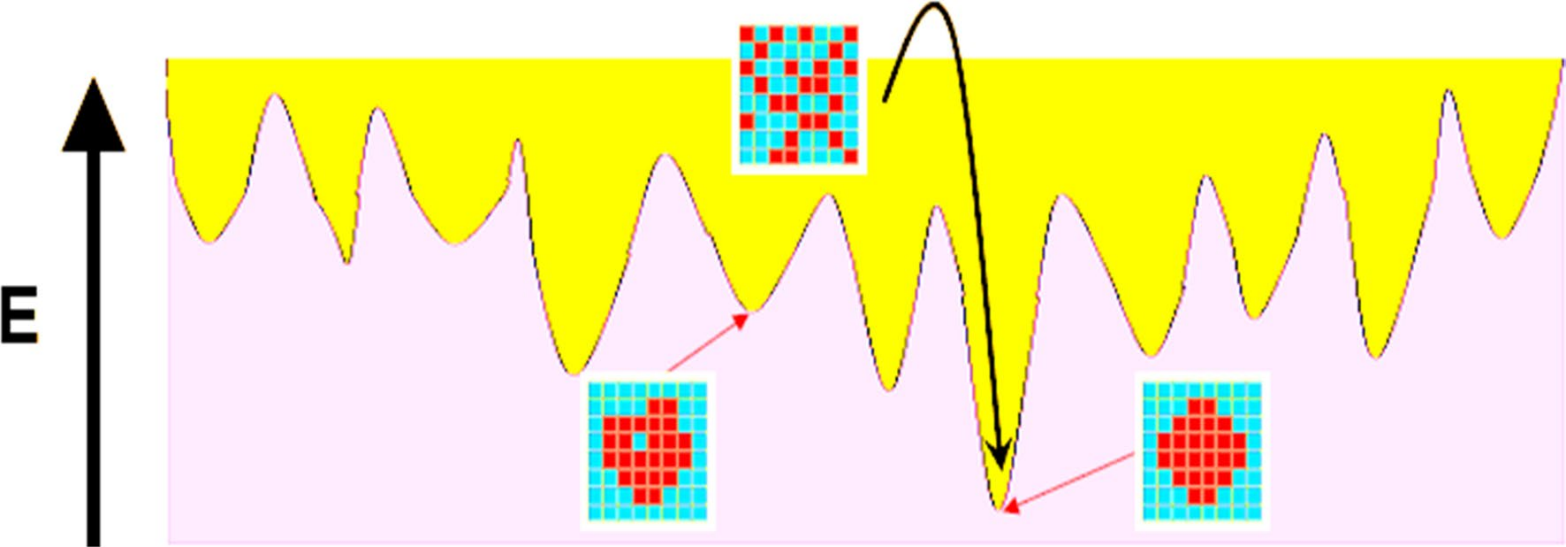
Visual representation of simulated annealing optimization procedure. The acceptance of energy-increasing trial microstructure allows the system to escape from local energy minima and thus, increases the probability of convergence to the global minimum.

Although, the modified annealing schedule has faster energy convergence, it puts the system at the risk of being trapped in local minima and optimum annealing is not guaranteed anymore, see Fig. 10.

Unfortunately, quantification of surface roughness via correlation function is not popular. We could not find any literature in such topic. There was no need to examine other surface-surface $F_{ss}$ or surface-void $F_{sv}$ functions that were defined in Ref.^12^ as the two-point correlation function $S_{2}$ was extremely efficient. Actually, representing surface roughness need to be studied for equal or higher than some distinct height. Note that it has higher impact on corrosion or effective general conductivity^16–18^. We expect that roughness cannot be studied as void-surface unless the void phase is taken as our matrix phase. That is why $S_{2}$ was our first choice to investigate the capability of roughness quantifications via statistical spatial correlation functions. On the following lines, we introduce the reconstruction using $S_{2} \left( r \right)$ of construction results as our target function.

**Figure 11:**
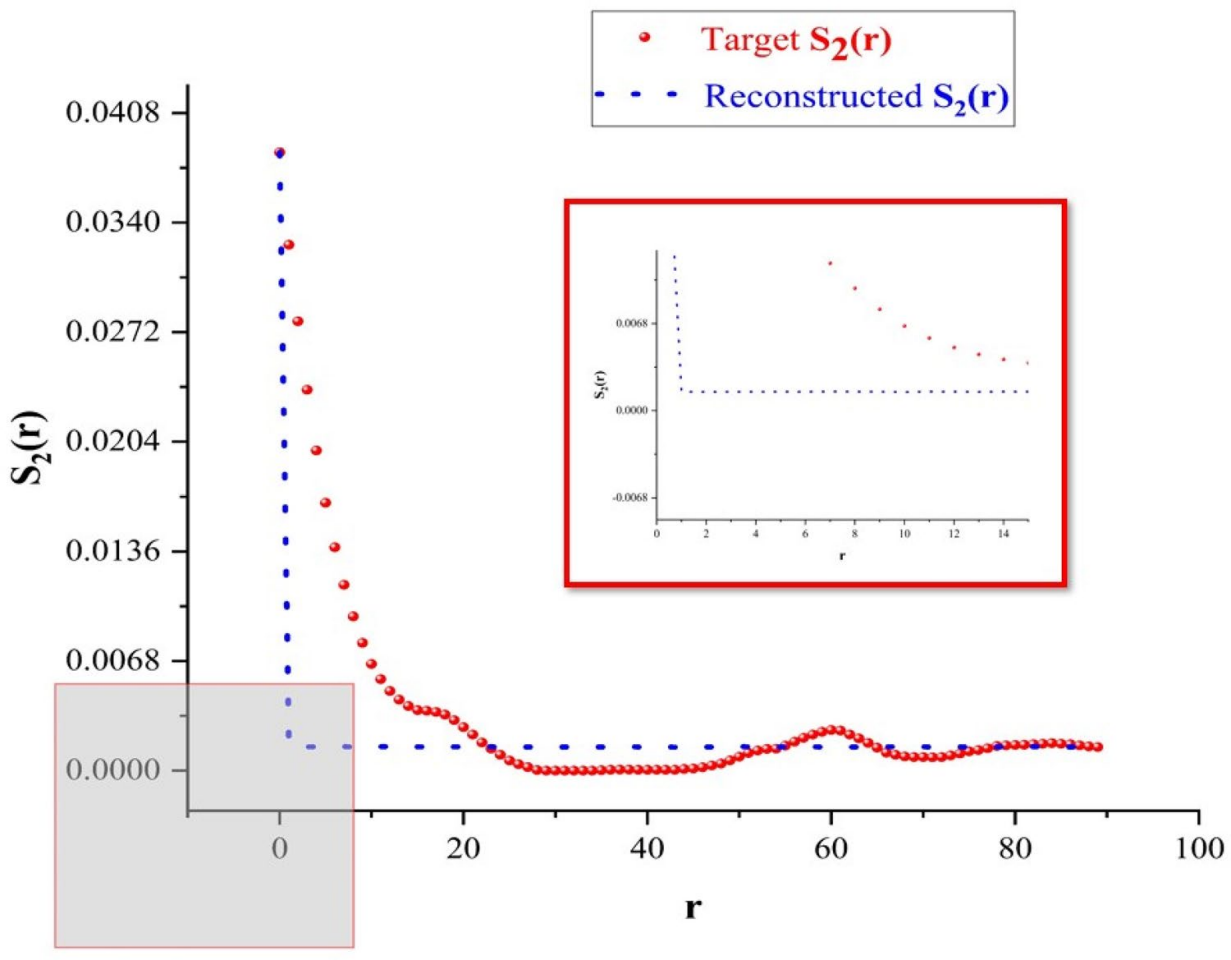
Reconstruction results upon using the two-point correlation function.

As shown in Fig. 11, the representation of surface roughness using the reconstruction of data obtained from the two-point correlation function, the optimization results in minimization of lengths of line segments of $S_{2} \left( r \right)$. As we take images for the surface at distinct heights (approximately 100 nm), line segment lengths are extremely decreased (${\text{average}} r \approx 0.001475)$, meaning that there is a propagation growth for the surface in horizontal directions, eliminating the surface roughness. This concludes that optimization of $S_{2} \left( r \right)$ is extremely efficient in smoothing the surface with very low rough lengths for such system.

## Conclusion

The possibility of quantifying surface roughness using low-order spatial correlation functions is demonstrated. Both two-point correlation function $S_{2} \left( r \right)$ and lineal-path function $L\left( r \right)$ were used to elucidate the surface characteristics of Pb–Sb alloy. This study demonstrated the importance of surface characteristics, such as roughness, on determining the performance of battery materials and their lifetime. Our study pointed out surface roughness as the main reason behind the observed battery failure upon the use of 2D additives to enhance the battery cyclability. The battery terminals were found to melt at a high discharge rate caused by poor heat transfer in the inner surface due to the high degree of roughness. The obtained results are very realistic and serve the aim of the study. The results demonstrate the opportunity of using two-point correlation functions to enhance the surface properties of a binary alloy by identifying certain roughness heights as the phase of interest. We conclude that this technique can also be applied in the metal polishing industry or for data mapping of the Pb–Sb alloy in the batteries industry.

## Data Availability

The datasets used and/or analysed during the current study available from the corresponding author on reasonable request.
